# Clinical outcomes after IL-6 blockade in patients with COVID-19 and HIV: a case series

**DOI:** 10.1186/s12981-022-00430-x

**Published:** 2022-02-11

**Authors:** Samuel J. Minkove, Grant Geiger, Josep M. Llibre, Mary W. Montgomery, Natalie E. West, Natasha M. Chida, Annukka A. R. Antar, Dima Dandachi, Ethel D. Weld, HIV-COVID-19 Consortium, Savannah Karmen-Tuohy, Philip M. Carlucci, Ioannis M. Zacharioudakis, Joseph Rahimian, Fainareti N. Zervou, Gabriel Rebick, Anna Stachel, Shini Tang, Dan Ding, Joyce L. Jones, Jason E. Farley, Kelly E. Dooley, Barbara E. Wilgus, Michael Sanchez, Jeremy Chow, Ellen Kitchell, Shannon Koh, Daniel Maxwell, Abby Lau, Shamika Brooks, Jessica Chu, Joshua Estrada, Susana M. Lazarte, Folasade Arinze, Adero Francis, Neha Paranjape, Paul E. Sax, Celestine N. Wanjalla, Asghar N. Kheshti, Samuel Bailin, John Koethe, Sean G. Kelly, Stephen P. Raffanti, Shital M. Patel, Teena Huan Xu, Melanie Goebel, Alberto Díaz-De Santiago, Manoj Ray, Jihad Slim, Ann Marie Porreca Kratz, David E. Koren, Kayla Hiryak, Brannon Hill, Ryan K. Dare, Stacie Bordelon, Brett Bailey, John W. Baddley, D. Matthew Shoemaker, Guillermo Rodriguez-Nava, F. N. U. Shweta, Carolyn Chu, Catherine Pearson, Amy Treakle, Jennifer J. Furin, Milana Bogorodskaya, Samit Desai, Danielle Osterholzer, Jered Arquiette, Emily S. Ford, Patrick R. Ching, Louisa Sun, Brian P. Buggy, Amir Tirmizi, Sarah Argentine, Balaji Desai, Talia H. Swartz, Dusty Latimer, Maraya Camazine

**Affiliations:** 1 Department of Medicine, The Johns Hopkins University School of Medicine, 600 N. Wolfe Street, Osler 508, Baltimore, MD 21287 USA; 2 Critical Care Medicine Department, National Institutes of Health, Bethesa, USA; 3 University of Missouri-Columbia School of Medicine, Columbia, USA; 4 Infectious Diseases and Fight AIDS Foundation, University Hospital Germans Trias, Badalona, Spain; 5 Department of Infectious Diseases, Brigham and Women’s Hospital, Boston, USA; 6 Department of Medicine, University of Missouri, Columbia, USA

## Abstract

**Background:**

In hospitalized people with HIV (PWH) there is an increased risk of mortality from COVID-19 among hospitalized PWH as compared to HIV-negative individuals. Evidence suggests that tocilizumab—a humanized monoclonal interleukin (IL)-6 receptor inhibitor (IL-6ri) antibody—has a modest mortality benefit when combined with corticosteroids in select hospitalized COVID-19 patients who are severely ill. Data on clinical outcomes after tocilizumab use in PWH with severe COVID-19 are lacking.

**Case presentation:**

We present a multinational case series of 18 PWH with COVID-19 who were treated with IL-6ri’s during the period from April to June 2020. Four patients received tocilizumab, six sarilumab, and eight received an undocumented IL-6ri. Of the 18 patients in the series, 4 (22%) had CD4 counts < 200 cells/mm^3^; 14 (82%) had a suppressed HIV viral load. Eight patients (44%), all admitted to ICU, were treated for secondary infection; 5 had a confirmed organism. Of the four patients with CD4 counts < 200 cells/mm^3^, three were treated for secondary infection, with 2 confirmed organisms. Overall outcomes were poor—12 patients (67%) were admitted to the ICU, 11 (61%) required mechanical ventilation, and 7 (39%) died.

**Conclusions:**

In this case series of hospitalized PWH with COVID-19 and given IL-6ri prior to the common use of corticosteroids, there are reports of secondary or co-infection in severely ill patients. Comprehensive studies in PWH, particularly with CD4 counts < 200 cells, are warranted to assess infectious and other outcomes after IL-6ri use, particularly in the context of co-administered corticosteroids.

**Supplementary Information:**

The online version contains supplementary material available at 10.1186/s12981-022-00430-x.

## Background

The interplay of COVID-19 and HIV is not completely understood [1]. However, a recent report by the World Health Organization (WHO) found that HIV was independently associated with a higher risk of in-hospital COVID-19 mortality, even after adjusting for age, sex, disease severity at admission, and a number of underlying conditions (adjusted Hazard Ratio (aHR) 1.30, 95% CI 1.24–1.36) [2]. Severe COVID-19 in the general population is associated with a hyperinflammatory syndrome similar to cytokine release syndrome (CRS) [3–5], thus, there have been several clinical trials of tocilizumab, an IL-6 receptor inhibitor (IL-6ri) approved for chimeric antigen receptor (CAR) T-cell-associated CRS [6–9]. One large randomized trial found that tocilizumab combined with corticosteroids led to a reduction in 28 day mortality in hypoxic patients with evidence of systemic inflammation (CRP ≥ 75 mg/L) [10]. Another trial found a reduction in organ support among ICU patients [9]. However, several smaller studies, particularly those enrolling before corticosteroids were in common use, have shown mixed results, and it remains unclear which patient population benefits most [11–14]. A meta-analysis of clinical trials of patients hospitalized for COVID-19 showed that treatment with an IL-6ri was associated with significantly lower 28-day all-cause mortality as compared to placebo or usual care (summary odds ratio (OR) 0.86 [95% CI 0.79–0.95]; p = 0.003) [15], and tocilizumab is now part of the National Institutes of Health (NIH) and Infectious Diseases Society of America COVID-19 treatment guidance for patients hospitalized with progressive or severe COVID-19 and elevated inflammatory markers [16, 17]. But, as use of an IL-6ri can be associated with increased risk of secondary infections [18–21], guidelines recommend against their use in severely immunocompromised patients [6].

It is unclear whether PWH have increased risk for secondary infections or other adverse events after the use of IL-6ri for severe COVID-19. There are few reports of PWH receiving IL-6ri for COVID-19 [22, 23]. Here, we present 4 narrative case reports accompanied by a description of the outcomes of 18 patients with a diagnosis of HIV and PCR-confirmed COVID-19 treated with IL-6ri between April and June 2020. As data was retrieved from a pre-existing data base (The COVID-19 in PWH Registry [24]) with a different clinical question, the specific IL-6ri could not always be known. Additionally, cases in this series occurred prior to the publication of the RECOVERY trial (June 16, 2020), which showed that dexamethasone lowered 28-day mortality among hospitalized patients with COVID-19 [8], and prior to FDA approval of remdesivir (October, 22 2020) [25]. Therefore, corticosteroids and remdesivir were not routinely used, and effects arising from the combination of these drugs and IL-6ri would not be accounted for.

## Methods

Data was combined from The COVID-19 in PWH Registry [24] and from internal chart review at the Johns Hopkins Hospital (JHU IRB00249576) between April and June 2020. The COVID-19 in PWH Registry is a multi-national registry (Additional file 1: Supplemental Methods) which is sponsored by the University of Missouri, Columbia (The study was reviewed by the University of Missouri Institutional Review Board and considered to be exempt MU IRB 00000731, 00009014).

## Results

Baseline characteristics from 18 PWH, including the 4 presented above, were collected across 8 centers in 3 countries between April 1, 2020 and June 30, 2020 and are presented in Table 1. Median age was 60.5 years (29–76), and thirteen (72%) patients were Black or Hispanic. At least 1 comorbid condition was present in 14 (78%) patients, with HTN, DM, and CKD most commonly reported. HIV VL was < 50 copies/mL in 14 (82%), unknown in 1, and > 200 copies/mL in 1 patient. CD4 counts were < 200 cells/mm^3^ in 4 (22%) patients, unknown for 1, and > 200 cells/mm^3^ in 13. All patients with previously diagnosed HIV were on antiretroviral therapy throughout their admissions; the patient with HIV/AIDS newly diagnosed during his ICU stay for COVID was never started on ART.

**Table 1:** Baseline characteristics of HIV patients receiving IL-6 inhibition for COVID-19 pneumonia

Pt	Sex	Age	Race/ethnicity	Location	Most recent VL	CD4	ART	Comorbid conditions
1	F	60	White, non-Hispanic	Int	Undetectable	600	2 NRTIs + PI + INSTI	COPD, CKD, Cirrhosis
2	M	39	Hispanic, white	Int	Undetectable	354	2 NRTIs + INSTI	HTN
3	M	74	Black, non-Hispanic	MA	Undetectable	238	2 NRTIs + INSTI	HTN, CKD, malignancy
4	F	76	Black, non-Hispanic	PA	Undetectable	205	2 NRTIs + INSTI	HTN, DM, Asthma, CKD
5	M	61	White, non-Hispanic	NY	29	62	2 NRTIs + INSTI + CYP3Ai + PI	Asthma
6	M	60	Hispanic; non-white	NY	Undetectable	1200	2 NRTIs + INSTI	None
7	M	59	Hispanic; non-white	NY	Undetectable	298	NNRTI + INSTI	HTN, DM
8	F	68	Hispanic, non-white	NY	Undetectable	186	2 NRTIs + INSTI	Cirrhosis
9	M	80	Black. non-Hispanic	NY	Undetectable	296	2 NRTIs + NNRTI	HTN, CKD
10	F	39	White, non-Hispanic	NY	Unknown	85	2 NRTIs + INSTI	None
11	M	29	Hispanic, non-white	NY	Undetectable	465	2 NRTIs + INSTI	HTN
12	M	36	Hispanic, white	TX	24	328	2 NRTIs + NNRTI	None
13	M	70	White, non-Hispanic	TX	Undetectable	260	2 NRTIs + INSTI	COPD, CKD
14	F	57	Black, non-Hispanic	PA	84	250	2 NRTIs + INSTI	HTN, DM, CKD
15	M	81	Hispanic, non-white	TX	91	345	2 NRTIs + PI	DM, malignancy
16	M	70	White, non-Hispanic	TX	Undetectable	260	2 NRTIs + NNRTI	COPD
17	M	61	Hispanic, non-white	NY	Undetectable	NR	2 NRTIs + INSTI	HTN, CAD
18	M	57	Black, non-Hispanic	MD	326	111	N/A	None

*Pt* patient, *Int* international, *MA* Massachusetts, *PA* Pennsylvania, *NY* New York, *TX* Texas, *MD* Maryland, *VL* viral load (1000 copies/mL), *CD4* most recent documented absolute CD4 count (cells/mm [3]), *COPD* chronic obstructive pulmonary disease, *CKD* chronic kidney disease, *HTN* hypertension, *DM* diabetes mellitus, *ART* antiretroviral therapy, *NRTI* nucleoside reverse transcriptase inhibitor, *NNRT* non-nucleoside reverse transcriptase inhibitor, *PI* protease inhibitor, *INSTI* integrase strand transfer inhibitor, *CYP3Ai* CYP3A inhibitor, *N/a* new diagnosis not on treatment

SARS-CoV2 was confirmed by PCR in all patients. The administered IL-6ri was tocilizumab in 4, sarilumab in 6, and an unknown IL-6ri in 8 patients (Table 2). Timing of administration was reported for 4 patients, all of them treated within 10 days of being hospitalized (3–10 days of hospitalization), and unknown for the remaining patients. Only 2 patients received corticosteroids, while none received remdesivir. On admission, median CRP was 104 (7–207) mg/L, with 9 (50%) patients > 100 mg/L. Median d-dimer was 0.26 (0–4) mg/mL—5 patients presented with d-dimer of ≤ 0.002 mg/mL, 12 > 0.18 mg/L, and 1 without a reported value. Presenting absolute lymphocyte count was < 1.0 × 10^3^ cells/µL in 7 (39%) patients. Secondary or opportunistic infections were treated in 8 (44%) patients, all admitted to the ICU, and 4 died. A specific organism was identified in 5 patients, including *Stenotrophomonas, S. aureus, C. albicans, E. cloacae, K. aerogenes* respiratory infections*,* and *disseminated VZV* (case 1). It was unclear whether these infections arose before or after IL-6ri in at least 3 of these cases. Of the 4 patients with CD4 counts < 200 cells/mm^3^, three were treated for secondary infection, with 2 confirmed organisms and 2 deaths, while in 13 patients with CD4 counts > 200 cells/mm^3^, 5 were treated for secondary infection, with 3 confirmed organisms and 5 deaths. Overall, ICU admission was required for 12 (67%) patients, including 3 of 4 subjects with CD4 counts < 200 cells, and 3 of 6 patients older than 65. Eleven (61%) patients required mechanical ventilation, and death occurred in 7 (39%) patients.

**Table 2:** Clinical course

Pt	IL-6 Inhibitor	Hospital day IL-6i administered	Corticosteroids	CRP*	d-Dimer	ALC (%)	Treated for secondary infection (confirmed organism)	ICU admission	Mechanical ventilation	Discharged alive
1	Tocilizumab	10	Y	21*	1100	1.1 (38)	No	No	No	Yes
2	Tocilizumab	3	Y	117*	542	1.1 (25)	No	No	No	Yes
3	Tocilizumab	9	N	94*	4000	1.0 (14)	No	No	No	No
4	NR*	NR**	N	15	1269	0.7 (10)	No	No	No	Yes
5	NR*	NR**	N	308	1103	0.3 (6)	Yes (unknown)	Yes	Yes	No
6	Sarilumab	NR**	N	68	226	1.7(32)	Yes (unknown)	Yes	Yes	Yes
7	NR*	NR**	N	188	184	0.1 (4)	Yes (S. maltophilia)	Yes	Yes	No
8	NR*	NR**	N	85	NR	0.7 (19)	No	No	No	Yes
9	NR*	NR**	N	134	1115	0.6 (5)	No	No	No	Yes
10	NR*	NR**	N	114	210	3.8 (34)	Yes (*S. aureus*)	Yes	Yes	Yes
11	NR*	NR**	N	190	264	1.3 (14)	Yes (*E. cloacae*)	Yes	Yes	Yes
12	Sarilumab	NR**	N	178	0	1.4 (13)	No	Yes	Yes	Yes
13	Sarilumab	NR**	N	21	2	1.2 (11)	No	Yes	Yes	No
14	Sarilumab	NR**	N	9	492	1.7 (29)	No	No	No	Yes
15	Sarilumab	NR**	N	26	2	0.7 (8)	Yes (k.aaerogenes, S. epidermidis)	Yes	Yes	No
16	Sarilumab	NR**	N	207	2	1.14 (11)	No	Yes	Yes	No
17	NR*	NR**	N	187	315	0.2 (1)	Yes (unknown)	Yes	Yes	Yes
18	Tocilizumab	4	N	7*	1	1.0 (8)	Yes (V. Zoster)	Yes	Yes	No

*Pt* patient, *CRP* C-reactive protein (mg/L), * = on admission, d-dimer (ng/mL), *Y* yes steroids administered for COVID treatment, *N* no steroids administered for COVID treatment, *ALC* absolute lymphocyte count (10^3^ cells/µL), (%) %lymphocyte, CRP, d-Dimer, ALC were all on admission, *MV* mechanical ventilation, *Int* international, NR* specific IL-6i not reported, but all received; NR** date of administration not reported

## Case presentations

### Case 1

A 57-year-old man with no known medical conditions presented to the emergency department (ED) with 1 week of fevers, dyspnea, and fatigue. He was hypoxic, requiring supplemental oxygen by nasal cannula (NC). SARS-COV-2 by PCR returned positive, ceftriaxone and azithromycin were initiated, and he was hospitalized. On admission IL-6 was 92 pg/mL (ref. < 10 pg/mL), CRP 7 mg/L (ref.: < 0.5 mg/L), and d-dimer 1 mg/L (ref.: 0.19–0.79 mg/L). On day 4 of hospitalization, hypoxemia progressed requiring intubation, and he received tocilizumab 480 mg, but not corticosteroids or remdesivir. Over the next 3 days he developed refractory hypoxemia and shock managed with prone positioning and vasopressors. Multisystem organ failure progressed, and he required continuous veno-venous hemodialysis. On day 8 of hospitalization, he developed transaminitis with AST 337 IU/L, ALT 242, alkaline phosphatase 181 that persisted. Additional infectious workup on Day 40 of hospitalization revealed a new diagnosis of AIDS (positive HIV-1 antibody, CD4 count 111 cells/mm^3^; HIV-RNA 326,000 copies/mL) and presumed late latent syphilis (positive anti-treponemal antibody, negative RPR, positive TPPA). He was treated with 3 once-weekly doses of Benzathine penicillin. After 47 days of hospitalization he was noted to have a diffuse papular rash which became vesicular, and was unroofed. PCR testing of these lesions was positive for Varicella Zoster Virus (VZV), confirming a diagnosis of disseminated VZV. Concurrently, the patient’s hypotension became refractory to vasopressors and maximal supportive measures, and care was ultimately withdrawn on hospital day 49. Autopsy was declined.

### Case 2

A 74-year-old man with known HIV (CD4 234 cells/mm^3^, VL undetectable, on Bictegravir/Emtricitabine/Tenofovir alafenamide), dementia, hypertension (HTN), stage 3 chronic kidney disease (CKD), and presumed gastric carcinoma treated with one dose of pembrolizumab, presented to the ED with abdominal pain, fatigue, and altered mental status. Presenting oxygen saturation was 97% on room air (RA) but worsened, requiring supplemental oxygen. He tested positive for SARS-CoV-2 by PCR. Initial computed tomography (CT) of the chest revealed bilateral opacities, pleural effusions, and presumed worsening metastatic spread of malignancy. On admission, IL-6 was 68 pg/mL, CRP 94 mg/L, and d-dimer 4 mg/mL. He was not eligible for remdesivir due to renal function; corticosteroids were not given. On day 9 of hospitalization, with persistent hypoxia and intermittent fevers, he received 400 mg tocilizumab. On day 16, hypoxia progressed, requiring oxygen via high flow nasal cannula (HFNC). A repeat CT scan revealed worsening pleural effusions. Given his cancer diagnosis and poor prognosis, he was transitioned to comfort measures the following day and died of multisystem organ failure on day 21 of his hospitalization.

### Case 3

A 60-year-old woman with prior intravenous drug use, HIV (CD4 600 cells/mm^3^, undetectable HIV VL, on Abacavir/Lamivudine + Raltegravir), recently cured hepatitis C virus, cirrhosis, and Stage 1 CKD presented to the ED with cough, headache, dyspnea, fevers, sore throat, myalgias, and fatigue. She was hypoxic, initially requiring 3–5 L/min of oxygen via NC, but soon escalated to Bilevel Positive Airway Pressure (BiPAP). Initial chest X-ray (CXR) revealed multifocal opacities, and contrasted chest CT was suggestive of viral pneumonia without a pulmonary embolism. SARS-CoV-2 RT-PCR was positive, she was initiated on ceftriaxone and azithromycin and hospitalized. On admission CRP was 21 mg/L, d-dimer 1.1 mg/mL, and absolute lymphocyte count 0.9 × 10^3^ cells/µL. She was treated with corticosteroids, but not remdesivir. On Hospital Day 2, she received 400 mg of tocilizumab and initiated 4 days of methylprednisolone and hydroxychloroquine. On day 8 of hospitalization, d-dimer peaked at 2023 ng/mL. Thereafter, she improved and was discharged to a continuing care facility on day 27 of her hospitalization with complete clinical recovery.

### Case 4

A 39-year-old man with HIV (CD4 354 cells/mm^3^, HIV VL undetectable, on Elvitegravir/Cobicistat/Emtricitabine/Tenofovir-alafenamide plus Darunavir 800 mg daily) and HTN presented with cough, dyspnea, fevers, sore throat, myalgias, and fatigue. In the ED, he was tachypneic, but saturating 96% on room air. SARS-CoV-2 was positive by RT-PCR. He was initiated on ceftriaxone, azithromycin, and hydroxychloroquine, and hospitalized. CXR revealed multifocal opacities, and on admission IL-6 was 73 pg/mL, CRP 117 mg/L, d-dimer 0.5 mg/mL, fibrinogen 984 mg/dL, and absolute lymphocyte count 0.8 × 10^3^ cells/µL. Over the next 2 days, hypoxia worsened and he required HFNC O_2_. IL-6 and d-dimer rose to 296 pg/mL and 843 ng/mL, respectively. On day 3 of hospitalization, he received 400 mg tocilizumab and initiated 4 days of methylprednisolone. He improved clinically and was discharged home on RA on hospital day 9.

## Discussion

Here we report one of the first case series of PWH who received IL-6ri for severe COVID-19, comprising 18 individuals. Most patients did not receive corticosteroids or remdesivir because of local standard of care in this time period early in the pandemic. 77% of the patients in this series had at least one co-morbid condition, increasing their risk for worse outcomes. The first diagnosis of HIV in a patient with AIDS at the time of ICU admission for COVID-19 (also previously reported) [22, 23] makes a compelling case for routine HIV screening on all patients hospitalized with severe or critical COVID-19.

All previously diagnosed patients were on anti-retroviral therapy, and HIV VL was < 50 copies/mL in 82% of patients, with CD4 counts over 200 cells/mm^3^ in 78% of patients. Still, there was a high prevalence of secondary infections. Although we are uncertain when the infections were present compared to administration of IL-6ri, clinical outcomes in the overall cohort and particularly among patients treated for secondary infection were poor, with death occurring in half of patients treated for infection. This is unsurprising given the baseline prognostic indicators, timing of this study during early pandemic surge [26], and the lack of contemporaneously available efficacious treatments such as corticosteroids and remdesivir [6].

As one of the only series of PWH receiving IL-6ri, this study adds a critical clinical perspective for providers treating PWH and COVID-19. The series draws from an ethnically diverse population from 8 hospitals with a high prevalence of co-morbid conditions. Confirmation of secondary infecting organisms was available for 64% of the patients treated for secondary infections, adding to an understanding of the diversity of secondary infections (along with COVID-19) which might behave opportunistically after treatment with IL-6ri.

As a case series, this series is retrospective, and the number of patients is few. Cases accrued prior to corticosteroids and remdesivir becoming standard of care for the treatment of COVID-19; only 2 patients received corticosteroids while none received remdesivir. Additionally, institutional policies on criteria and timing of administration of IL-6ri varied widely by site, and were not always clearly documented. Finally, data were obtained from a database that did not include specification of which exact IL-6ri was used, and this information was therefore not always obtainable. As head-to-head comparisons of one IL-6ri verses another are scarce, the impact of this limitation on the results is unclear.

## Conclusion

There is currently a lack of data on how best to individualize care for PWH with COVID-19. Immune-based therapy for COVID-19, while potentially lifesaving, may have potential for harm in certain immunocompromised patients, such as PWH with CD4 < 200 cells/mm^3^ or AIDS. Given the tens of millions of PWH living in settings with poor access to COVID-19 vaccines, this series highlights the need for further high-quality investigations of IL-6ri in PWH who are hospitalized with COVID-19.

## Supplementary Information

**Additional file 1**: Supplementary methods

## Data Availability

Raw data were generated from The COVID-19 in PWH Registry [24] and from internal chart review at the Johns Hopkins Hospital (JHU IRB00249576). Derived data supporting the findings of this study are available from the corresponding author EDW on request.
