# Supplementary material for: Clinical outcomes after IL-6 blockade in patients with COVID-19 and HIV: a case series
Source: AIDS Res Ther. 2022 Feb 11;19:6. doi: 10.1186/s12981-022-00430-x (PMC8832430; doi:10.1186/s12981-022-00430-x)
Supplement: Supplementary file 1 — Additional file 1: Supplementary methods [file 12981_2022_430_MOESM1_ESM.docx]

**Supplemental Methods: The COVID-19 in PWH Registry**

**Study Design**

This multicenter registry was for PWH who had COVID-19 and received care between 1 April and 1 July 2020. The study was listed on ClinicalTrials.gov (NCT04333953) and the Infectious Diseases Society of America (IDSA) website and was open to enrollment in the United States and internationally. The registry was promoted in the IDSA and HIV Medical Association discussion forums, and invitation emails were sent to Infectious Disease departments and HIV clinics across the United States. Patients aged 18 years and older with a known diagnosis of HIV and laboratory-confirmed COVID-19 in both inpatient and outpatient settings were eligible for study inclusion. Health-care providers collected study data by chart review of PWH with COVID-19 diagnosed at their facilities, and entered anonymous information into a secure, electronic Research Electronic Data Capture (REDCap) system. Patients were enrolled consecutively by nonrandom sampling. Study variables included patient demographics, HIV-associated variables, underlying medical problems, COVID-19 clinical presentation as reported by patients, laboratory values, treatment, and clinical outcomes. Providers certified that the information submitted was accurate to the best of their knowledge. The data were cross-validated by 2 reviewers for duplicity by age, gender, race, location, and HIV-1 RNA (viral load).

**Study Definitions**

Laboratory-confirmed COVID-19 was defined as positive reverse-transcriptase polymerase chain reaction (RT-PCR) in respiratory samples or serum SARS-CoV-2–specific immunoglobin G or M. The US geographical region of residence was based on the Centers for Disease and Prevention’s National HIV Surveillance System region distribution. Chronic lung disease included asthma and chronic obstructive pulmonary disease. Cardiovascular disease included coronary artery disease and congestive heart failure. Chronic liver disease included cirrhosis, chronic hepatitis B, and chronic untreated hepatitis C. Active malignancy excluded nonmelanoma skin cancer. We defined obesity as a body mass index ≥30. We defined virologic suppression as HIV-1 RNA (viral load) <200 copies/mL. We collected the most recent HIV viral load measured before or at the time of COVID-19 presentation. ART categorizations were mutually exclusive.

**IL-6 Administration**

IL-6 administration was unblinded and off label at the time of this series. Decisions on administration was based either on hospital or institutional protocol or based on clinician preference. Policies varied widely and are not well captured by this database.
